# Similar immune mechanisms control experimental airway eosinophilia elicited by different allergens and treatment protocols

**DOI:** 10.1186/s12865-019-0295-y

**Published:** 2019-06-04

**Authors:** Evelyn J. Hyde, Kirsty A. Wakelin, Naomi J. Daniels, Sayani Ghosh, Franca Ronchese

**Affiliations:** Malaghan Institute of Medical Research, Wellington, 6021 New Zealand

**Keywords:** Allergic airway inflammation, Innate immunity, Adaptive immunity, OVA, HDM

## Abstract

**Background:**

Mouse models have been extremely valuable in identifying the fundamental mechanisms of airway inflammation that underlie human allergic asthma. Several models are commonly used, employing different methods and routes of sensitisation, and allergens of varying clinical relevance. Although all models elicit similar hallmarks of allergic airway inflammation, including airway eosinophilia, goblet cell hyperplasia and cellular infiltration in lung, it is not established whether they do so by involving the same mechanisms.

**Results:**

We compared the impact of inactivation of various innate or adaptive immune genes, as well as sex, in different models of allergic airway inflammation in mice of C57BL/6 background. Chicken ovalbumin (OVA) and house dust mite (HDM) were used as allergens in settings of single or multiple intranasal (i.n.) challenges, after sensitisation in adjuvant or in adjuvant-free conditions. Eosinophil numbers in the broncho-alveolar lavage and lung histopathology were assessed in each model. We found that Major Histocompatibility Complex Class II (MHCII) deficiency and lack of conventional CD4+ T cells had the most profound effect, essentially ablating airway eosinophilia and goblet cell hyperplasia in all models. In contrast, Thymic stromal lymphopoietin receptor (TSLPR) deficiency greatly reduced eosinophilia but had a variable effect on goblet cells. CD1d deficiency and lack of Natural Killer T (NKT) cells moderately impaired inflammation in OVA models but not HDM, whereas sex affected the response to HDM but not OVA. Lastly, defective Toll-like receptor (TLR)4 expression had only a relatively modest overall impact on inflammation.

**Conclusion:**

All the models studied were comparably dependent on adaptive CD4+ T cell responses and TSLP. In contrast, sex, NKT cells and TLR4 appeared to play subtler and more variable roles that were dependent on the type of allergen and mode of immunization and challenge. These results are consistent with clinical data suggesting a key role of CD4+ T cells and TSLP in patients with allergic asthma.

**Electronic supplementary material:**

The online version of this article (10.1186/s12865-019-0295-y) contains supplementary material, which is available to authorized users.

## Background

Allergic disease is caused by the inappropriate activation of Th2 cells in response to harmless or non-infectious stimuli such as pollens, foods or insect stings. In allergic individuals, Th2 recognition of allergens in the context of antigen presenting cells triggers the release of cytokines, including Interleukin (IL)-3, IL-4, IL-5 and IL-13, which underlie the typical allergic pathology with recruitment of mast cells and eosinophils, mucus production, IgE and tissue remodeling [1].

In the airway, allergic disease is elicited by allergen inhalation and manifests itself with the typical asthma symptoms of tightness of chest and reduced lung function. Broncho-alveolar lavage of segmentally challenged allergic asthma patients has revealed the presence of eosinophils and Th2 cells in the airway with increased mucin, Th2 cytokines and increased smooth muscle mass [2]. Animal models of allergic airway inflammation faithfully replicate several of these hallmarks of the late asthmatic response: type 2-associated cytokines such as IL-4, IL-5 and IL-13 can be found in the broncho-alveolar lavage (BAL) and are responsible for the observed inflammatory pathologies [3, 4].

Chicken ovalbumin (OVA) has been used extensively as a model allergen, proving valuable in elucidating many features of airway disease. OVA is inexpensive and readily available, with well-characterized MHCI and MHCII epitopes. In addition, the availability of the OVA-specific OTI and OTII T cell receptor transgenic mice greatly facilitates the monitoring of OVA-specific immune responses in airways and local lymph nodes [5]. These features have made OVA a reagent of choice when studying the cellular mechanisms underlying airway inflammatory responses. However, although OVA is not a completely irrelevant allergen, as food allergies to egg are relatively common in humans [6], it is not clinically relevant as an airway allergen. In addition, OVA is not naturally immunogenic upon inhalation, and can induce tolerance [7] unless supplemented with low doses of LPS [8]. In order to sensitise to allergic airway inflammation, OVA is normally used adsorbed to the adjuvant aluminium hydroxide and must be administered via the non-physiological i.p. route, thus bypassing the airway innate immune environment during sensitisation [9]. Due to these drawbacks, the clinical relevance of information obtained using the OVA model has been questioned by some investigators.

Other allergens of higher clinical relevance being used in experimental airway allergy models include the house dust mite (HDM) *Dermatophagoides pteronyssinus*, cockroach, and the fungus *Alternaria alternata*. These allergens can sensitise mice when given via i.n. instillation without adjuvant, and indeed asthma patients are commonly atopic against one or more of them. One of their common features is the harbouring of innate properties, such as protease activity that can directly affect lung epithelium and other cell populations [10, 11], or the ability to engage Toll-like receptors (TLR) through protein mimicry [12], thereby eliciting epithelial cell production of alarmins and cytokines such as IL-33, thymic stromal lymphopoietin (TSLP) and IL-25 [13]. These cytokines act on multiple cell types including dendritic cells (DC) and pathogenic Th2 cells [14], and can also induce the production of IL-5 and IL-13 from local innate immune cell populations such as type-2 innate lymphoid cells (ILC2) independently of conventional Th2 cells [15–17]. Therefore, the innate properties of allergens may have a substantial impact on the cell populations involved in allergic responses in different models.

In this study, we wished to assess to what degree the choice of allergen can affect the mechanism of allergic airway inflammation in different experimental models. We compared four models, which use the allergens OVA or HDM and employ different protocols of allergen sensitisation and challenge, focusing on airway eosinophilia and goblet cell hyperplasia as these responses can be elicited by either ILC2 or CD4+ Th2 cells. We compared these models in different strains of knockout (KO) mice that are defective in selected components of the adaptive or innate immune response, and found that, in most cases, allergic inflammation was comparably reduced in each KO strain across all four models, with the degree of reduction ranging from mild to strong. Our results reveal notable similarities, but also some subtle differences, in the molecules and cell types driving each model, thus suggesting the involvement of similar immunological mechanisms in each case.

## Results

### Airway eosinophilia and goblet cell hyperplasia can be induced in mice using several protocols of allergen immunization and challenge

**Fig. 1:**
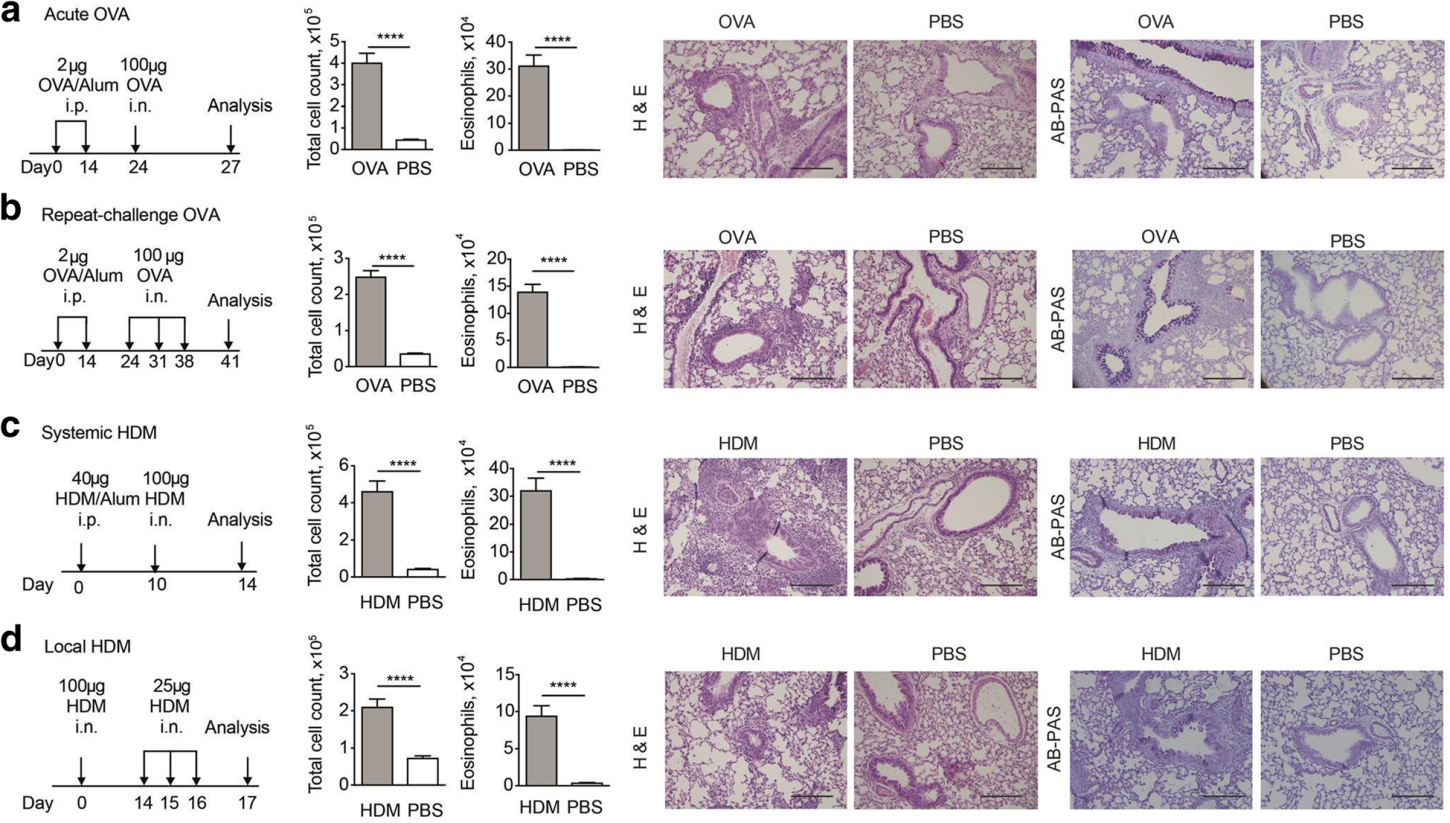
Features of allergic airway inflammation induced using different models. (**a**–**d**) Mice were treated according to the protocols shown on the left; PBS mice were sensitised with PBS/alum, or PBS only for the local HDM model. Five to seven experiments were performed for each model, for a total of 21–31 mice per group. Bar graphs show mean + SEM of the combined results from all experiments in each model; please note that y-axes differ across models. Representative micrographs of lung histology are taken from mice with eosinophil numbers similar to the mean. Scale bars represent 200 μm. ***, *p* < 0.001; **, *p* < 0.01; *, *p* < 0.05; ns, not significant

We aimed to compare the role of key immunological mechanisms in various models of allergic airway inflammation that are commonly used in the literature. To this end, we selected two models using OVA as the model allergen [18]. Both models utilize an identical protocol of sensitisation in which 2 μg of OVA adsorbed to alum is administered intraperitoneally at days 0 and 14. As a negative control, phosphate-buffered saline (PBS) and alum were used during sensitisation, whilst the airway challenges remained the same. In the “acute” model (Fig. 1a), a single challenge of low-endotoxin OVA was administered i.n. 10 days after the second sensitisation. In the “repeat challenge” model (Fig. 1b), three i.n. instillations were applied at one-week intervals. In both models, BAL was harvested 3 days after the final challenge to analyse inflammatory cell populations in the airway by flow cytometry. In addition, lungs were collected at endpoint to document histological changes. In both models we observed higher frequency and number of eosinophils in BAL, and higher numbers of neutrophils and T cells (Additional file 1) which did not increase, or even decreased, with multiple i.n. challenges. Development of peribronchial and perivascular inflammation in the lungs (Fig. 1) and PAS-positive staining in the bronchiolar epithelium (Fig. 1, with quantification as percent of PAS-positive staining in epithelium in Additional file 2B) were observed in both models. Unlike eosinophil numbers, PAS-positive staining increased with multiple challenges.

We also used two models of allergic airway inflammation with HDM as the allergen. In the systemic sensitisation model [19], HDM extract or PBS in alum were injected i.p. once, followed 10 days later by a single i.n. challenge of the same HDM extract (Fig. 1c). BAL and lung tissue were harvested for analysis 4 days after challenge, revealing a marked eosinophil infiltrate in the airway, extensive peribronchial and perivascular inflammation, and PAS-positive staining in the bronchiolar epithelium (Additional file 2B). Neutrophils and T cells were also elevated (Additional file 1).

In the local sensitisation model (Fig. 1d), HDM or PBS were given by i.n. instillation without adjuvant, to mimic the natural exposure to airborne allergens via the nose and airway tract. Sensitisation was followed by three HDM i.n. challenges on days 14–16 [20], with BAL and tissue harvest on day 17. Compared to other models, the number of total BAL cells was lower in the immunised group, and higher in the PBS controls. Total eosinophils in the airway were clearly increased compared to control mice, although lower than in other models. Neutrophil numbers were highest in this model, likely due to BAL being carried out the next day after the last HDM challenge [21]. In contrast, T cells were lowest (Additional file 1). PAS-positive staining in bronchiolar epithelium and cellular infiltration in the lung were also detectable to similar levels as in the other models (Fig. 1d and Additional file 2B).

Thus, each of the four models successfully elicited eosinophil accumulation in the airway, goblet cell hyperplasia in bronchioles, and immune cell infiltration in lung tissue.

### MHCII-KO mice are refractory to airway eosinophilia and goblet cell hyperplasia

**Fig. 2:**
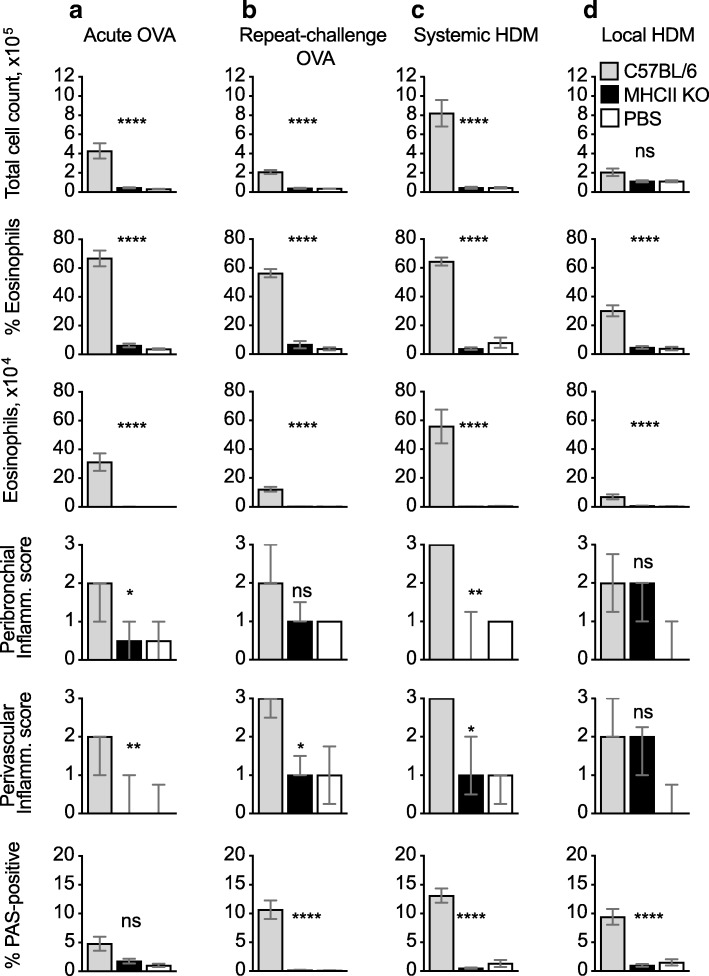
MHCII-KO mice fail to generate allergic airway inflammation to OVA or HDM. (**a**–**d**) C57BL/6 and MHCII-KO mice were immunized and challenged with OVA or HDM as in Fig. 1, PBS refers to C57BL/6 mice that were mock-immunized and challenged with OVA or HDM. Total cell counts, percent and number of eosinophils were evaluated by flow cytometry; bar graphs show mean + SEM/mouse from two combined experiments each using 5–8 mice per group. Histopathological scores were calculated on 6–10 mice per condition, selected from both experiments. Peribronchiolar and perivascular inflammation scores are displayed as median +/− interquartile range; the percent PAS-positive staining in bronchiolar epithelial cells is shown as mean + SEM. *P* values refer to comparisons between the sensitised and challenged C57BL/6 and MHCII-KO groups. ***, *p* < 0.001; **, *p* < 0.01; *, *p* < 0.05; ns, not significant

To assess the relative role of CD4+ T cells vs. innate cell types in our models, we utilized MHCII-KO mice which express undetectable levels of MHCII and very few conventional CD4+ T cells [22]. We tested the response of these mice to induction of allergic airway inflammation in the four models described in Fig. 1. In each case we observed that total BAL cellularity, as well as frequency and number of eosinophils were very low and equal to background (Fig. 2a-d). Neutrophil and T cell numbers were also lower than in C57BL/6 controls, except for the local HDM model (Additional file 3). Peribronchial and perivascular inflammation were reduced to background levels, with the only exception of the local HDM model in which a moderate inflammation was observed in both C57BL/6 and MHCII-KO mice. PAS-positive staining in bronchiolar epithelium was not increased compared to PBS in all models (Fig. 2).

Therefore, in all models tested, eosinophilic inflammation and goblet cell hyperplasia showed a complete requirement for conventional CD4+ T cells, whereas lung cellular infiltration was only partly affected.

### TSLPR-KO mice develop reduced eosinophilia across all models

**Fig. 3:**
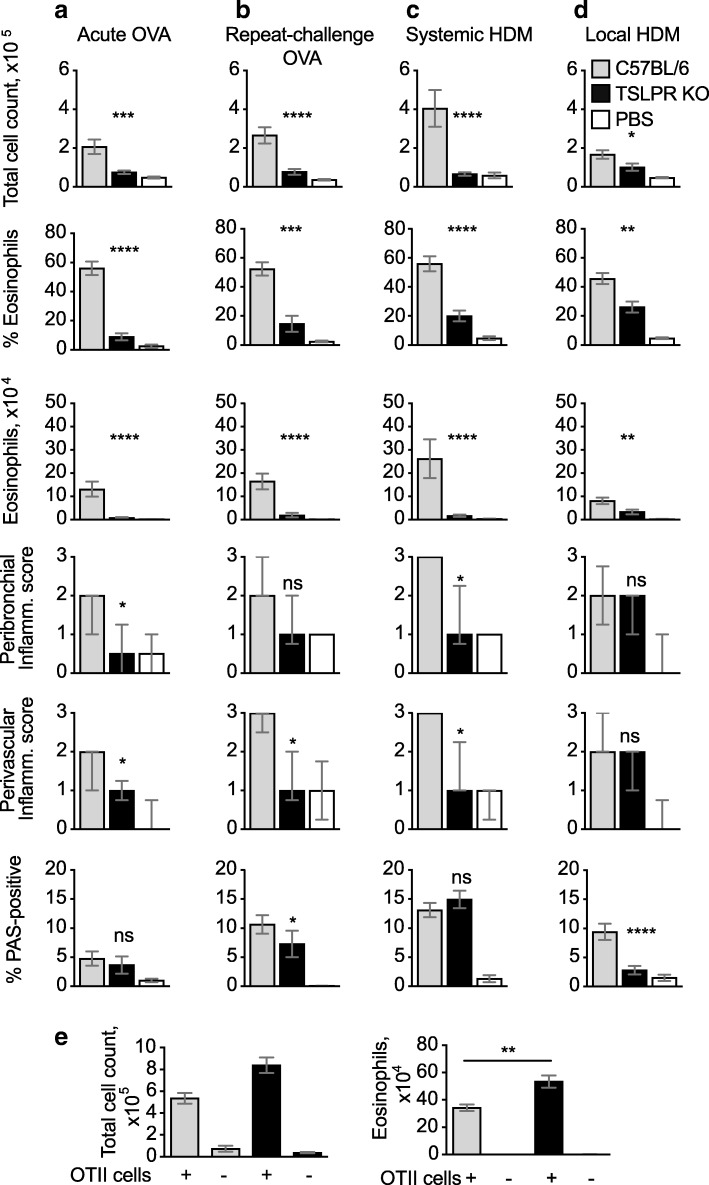
TSLPR deficiency results in reduced airway inflammation in OVA and HDM models. (**a**–**d**) C57BL/6 and TSLPR-KO mice were immunized and challenged with OVA or HDM as in Fig. 1; PBS refers to C57BL/6 mice that were mock-immunized and challenged with OVA or HDM. Total cell counts, percent and number of eosinophils were evaluated by flow cytometry; bar graphs show mean + SEM/mouse from two combined experiments each using 5–8 mice per group. Histopathological scores were calculated on 6–10 mice per condition, which were selected from both experiments. Peribronchiolar and perivascular inflammation scores are displayed as median +/− interquartile range; the percent PAS-positive staining in bronchiolar epithelial cells is shown as mean + SEM. (**e**) In vitro activated OTII Th2 cells were transferred into WT or TSLPR-KO mice 1 day before i.n. OVA challenge; data refer to 6–7 mice per group. In A-D, P values refer to the comparison between the sensitised and challenged C57BL/6 and TSLPR-KO groups. ***, *p* < 0.001; **, *p* < 0.01; *, *p* < 0.05; ns, not significant

TSLP is a key cytokine in Th2 immune responses, affecting multiple cell populations including DC, ILC2 and CD4+ Th2 cells [23]. We compared the responses of C57BL/6 and TSLPR-KO mice in the four models of allergic airway inflammation using OVA or HDM. We found that in all cases total cellularity and eosinophil percentages and numbers in BAL were greatly reduced in TSLPR-KO mice compared to WT (Fig. 3a–d). Neutrophils and T cells were also decreased, except in the local HDM model (Additional file 3).

Other parameters of airway inflammation were also compared: lung histology revealed moderately reduced peribronchial and perivascular inflammation in TSLPR-KO mice compared to WT in all models (Fig. 3a-c), with the exception of the local HDM model where the response was not affected (Fig. 3d). PAS-positive staining in bronchiolar epithelial cells was normal or slightly reduced in TSLPR-KO mice compared to WT in all models (Fig. 3a-c), with the exception of the local HDM model where a significant reduction was noted (Fig. 3d).

To assess whether TSLP was necessary for the function of CD4+ Th2 cells or also other cell populations, TSLPR-sufficient OTII cells were activated in vitro in Th2 conditions and transferred into WT C57BL/6 or TSLPR-KO recipients 1 day before OVA challenge. Eosinophil numbers were not reduced in TSLPR-KO mice, or were even slightly exacerbated compared with WT mice (Fig. 3e).

Overall, we observed a strong decrease in eosinophil numbers in TSLPR-KO mice in all models of allergic inflammation, whereas the effect on lung histology was mild or not detectable. In no case was the effect as marked as in MHCII-KO mice.

### CD1d-KO mice show impaired airway eosinophilia in responses to OVA allergen, but not HDM

NKT cells are innate-like lymphocytes that are capable of responding rapidly to stimuli including endogenous and exogenous glycolipids presented on CD1d molecules, and can mediate allergic airway inflammation independently of conventional CD4+ T cells [16].

**Fig. 4:**
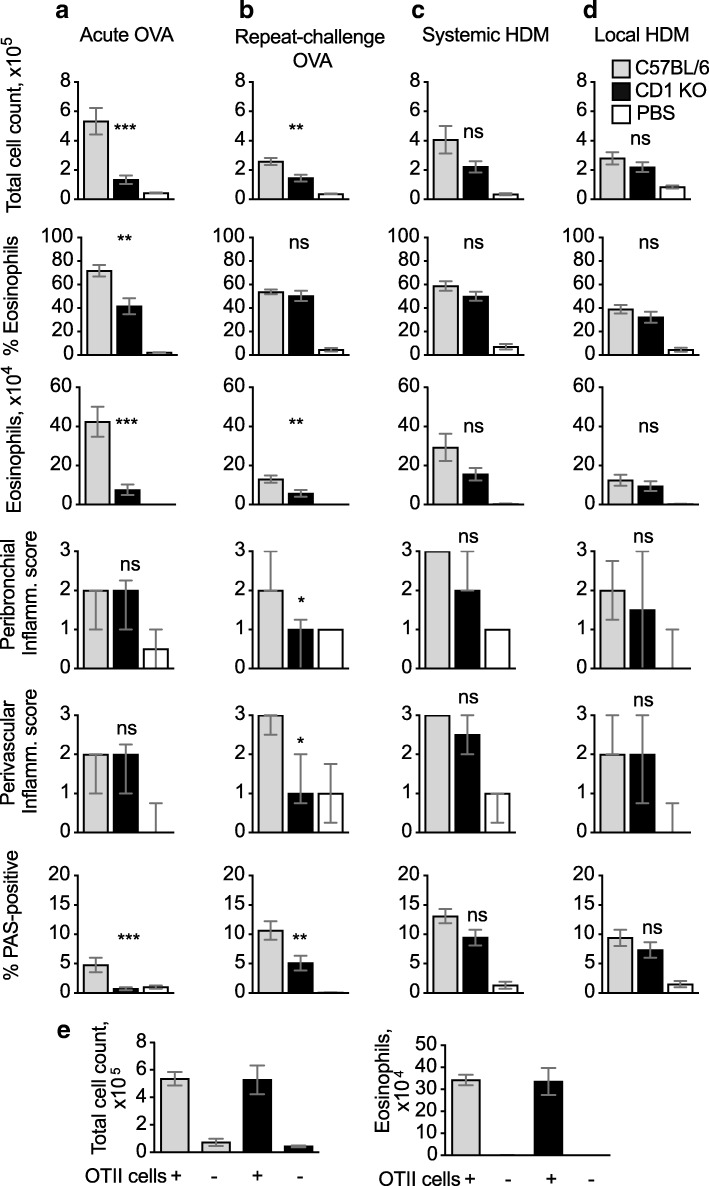
CD1d-KO mice generate reduced airway inflammation in OVA but not HDM models. (**a**–**d**) C57BL/6 and CD1d-KO mice were immunized and challenged with OVA or HDM as in Fig. 1; PBS refers to C57BL/6 mice that were mock-immunized and challenged with OVA or HDM. Total cell counts, percent and number of eosinophils were evaluated by flow cytometry; bar graphs show mean + SEM/mouse from two combined experiments each using 5–8 mice per group. Histopathological scores were calculated on 6–10 mice per condition, which were selected from both experiments. Peribronchiolar and perivascular inflammation scores are displayed as median +/− interquartile range; the percent PAS-positive staining in bronchiolar epithelial cells is shown as mean + SEM. (**e**) In vitro activated OTII Th2 cells were transferred into WT or CD1d-KO mice 1 day before i.n. OVA challenge; data refer to 6–7 mice per group. In A-D, P values refer to the comparison between the sensitised and challenged C57BL/6 and CD1d-KO groups. ***, *p* < 0.001; **, *p* < 0.01; *, *p* < 0.05; ns, not significant

We used CD1d-KO mice, which lack NKT cells, to assess the development of airway inflammation in our models. In both the acute and repeat-challenge OVA models, total cell counts and eosinophil numbers were lower in CD1d-KO mice compared to C57BL/6, whereas the frequencies of eosinophils were similar to WT or only moderately decreased (Fig. 4a, b). In addition, numbers of T cells were lower than in C57BL/6 controls in the acute OVA model (Additional file 3). PAS-positive staining of epithelial cells in bronchioles was also lower in CD1d-KO mice compared to WT, whereas inflammation scores were decreased in the repeat-challenge OVA model, but not in the acute one.

In contrast to the OVA models, the systemic and local HDM models were not significantly affected by lack of NKT cells (Fig. 4e, d). There was no statistically significant reduction in total or eosinophil BAL cellularity, although there was a trend towards lower eosinophil numbers in the CD1d-KO mice. Accumulation of inflammatory cells in the peribronchial and perivascular spaces was not affected by lack of NKT cells. PAS-positive staining in bronchiolar epithelial cells was also similar in control and CD1d-KO mice.

To assess whether in the acute OVA model NKT cell activity was required at the time of sensitisation or during airway challenge, we used adoptive transfer of OTII cells that were activated in vitro in Th2 conditions and injected into WT or CD1d-KO mice. I.n. challenge resulted in similar cellularity and eosinophil numbers in CD1d-KO and C57BL/6 mice (Fig. 4e), suggesting that NKT cell activity was not required at the time of OVA challenge.

Overall, lack of NKT cells resulted in a reduction of allergic airway inflammation in OVA models, whereas inflammation in the HDM models was essentially normal.

### TLR4 deficiency does not impair airway eosinophilia, goblet cell hyperplasia, or inflammatory infiltrate in mice exposed to OVA or HDM

**Fig. 5:**
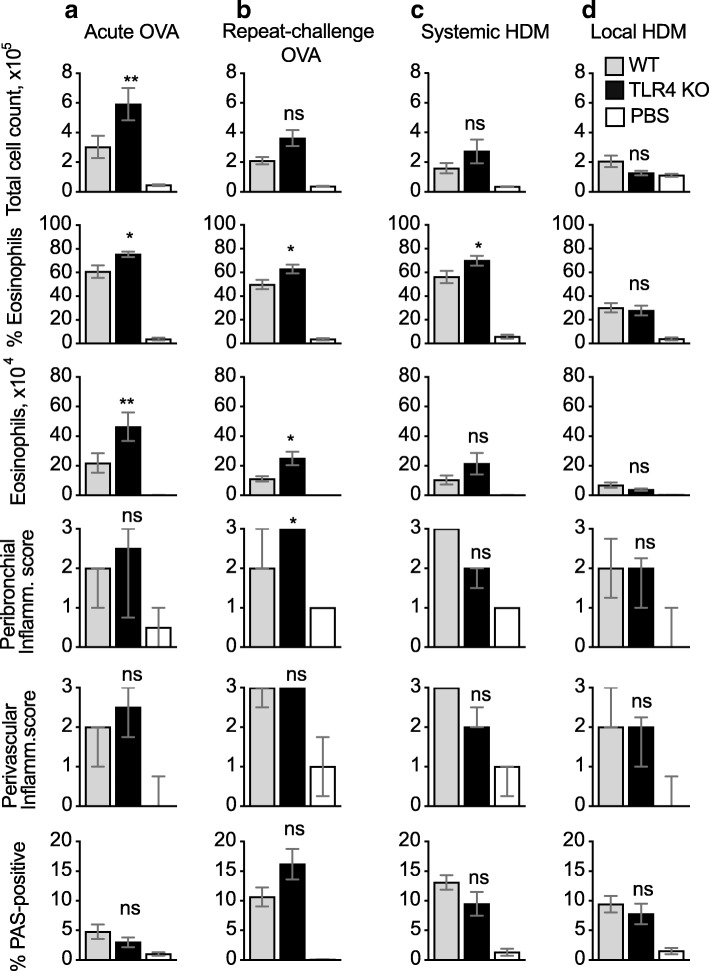
TLR4 deficiency mildly increases airway eosinophil numbers in OVA, but not HDM, models. (**a**–**d**) C57BL/6 and TLR4-KO mice were immunized and challenged with OVA or HDM as in Fig. 1; PBS refers to C57BL/6 mice that were mock-immunized and challenged with OVA or HDM. Total cell counts, percent and number of eosinophils were evaluated by flow cytometry; bar graphs show mean + SEM/mouse from two combined experiments each using 5–8 mice per group. Histopathological scores were calculated on 6–10 mice per condition, which were selected from both experiments. Peribronchiolar and perivascular inflammation scores are displayed as median +/− interquartile range; the percent PAS-positive staining in bronchiolar epithelial cells is shown as mean + SEM. P values refer to the comparison between the sensitised and challenged C57BL/6 and TLR4-KO groups. ***, *p* < 0.001; **, *p* < 0.01; *, *p* < 0.05; ns, not significant

We used TLR4-KO mice to compare the role of TLR4 in different models of airway inflammation. TLR4-KO mice exposed to the acute and repeat-challenge OVA models developed an intact or even slightly increased inflammatory response (Fig. 5a, b). As the OVA preparation used for i.n. challenge was low in endotoxin content, these effects may be due to endotoxin at the priming stage, or from other sources including the environment. Regardless of the endotoxin source, these experiments suggest that a low level of TLR4 signaling can limit inflammation in the OVA models in WT mice.

TLR4-KO mice exposed to systemic or local HDM generated eosinophil responses that were either similar to those in C57BL/6 mice, or marginally increased in both percentage and number (Fig. 5c, d). Perivascular and peribronchial inflammation scores, and the PAS-positive staining in bronchiolar epithelial cells, were not affected.

Overall, TLR4 deficiency did not appear to substantially affect the response to OVA or HDM allergens in these models.

### Female and male mice generate similar airway eosinophilia to OVA but not HDM

Previous studies in BALB/c mice have reported a stronger susceptibility of female mice to airway inflammation due to increased numbers of ILC2 [24]. However, it is currently unclear whether this difference extends to other mouse strains such as C57BL/6, and different allergens and exposure protocols.

**Fig. 6:**
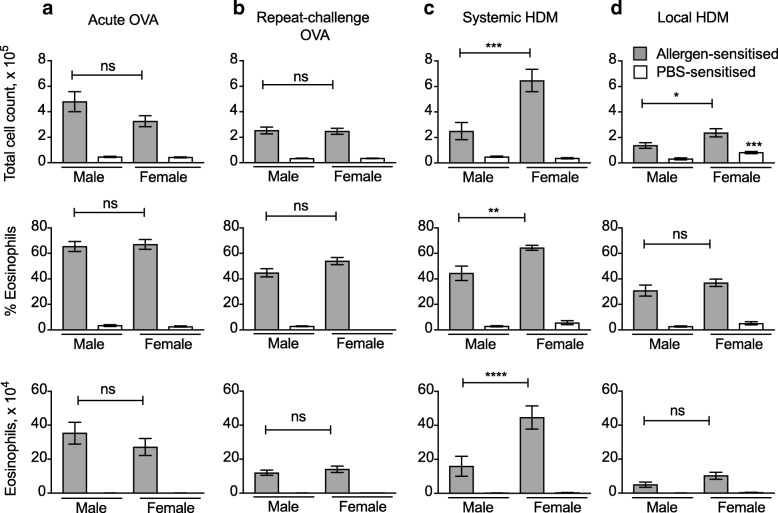
Female mice develop exacerbated airway eosinophilia in systemic HDM models compared to males. (**a**–**d**) Male and female C57BL/6 mice were immunized and challenged with OVA or HDM as in Fig. 1; PBS mice were mock-immunized and challenged with OVA or HDM. Total cell counts, percent and number of eosinophils were evaluated by flow cytometry; bar graphs show mean +/− SEM/mouse from several combined independent experiments each using 5–8 mice per group. P values refer to the comparison between males and females within sensitised and challenged groups, or mock-sensitised and challenged groups. ***, *p* < 0.001; **, *p* < 0.01; *, *p* < 0.05; ns, not significant

We combined all experiments carried out as part of this and other studies in our Laboratory, to compare C57BL/6 female and male mice for their ability to generate airway eosinophilia after allergen challenge. As shown in Fig. 6a and b, BAL cellularity, and also the number and percent of airway eosinophils, were similar in female and male mice undergoing the acute or repeat-exposure OVA protocols. In contrast, treatment with the systemic HDM protocol induced a stronger response in female mice, with about twice as many airway eosinophils in females compared to males (Fig. 6c). A trend to higher responses in female mice was also noted using the HDM local protocol, however, these differences were small and reached significance only in the case of total cellularity (Fig. 6d). PBS-sensitised male and female mice gave similar responses in all cases, with the exception of the total cell count in the local HDM model (*p* < 0.001).

Thus, a comparison of male and female C57BL/6 mice revealed differences in the degree of airway eosinophilia that were dependent on both the type of allergen and the treatment protocol.

## Discussion

We used four models of allergic airway inflammation that employ different allergens (OVA and HDM) priming protocols (systemic vs. local) and airway challenges (acute vs. repeated), to compare the contribution of four key immune response genes; MHCII, TSLPR, CD1d and TLR4, to various parameters of airway inflammation in each of the models. Differences between C57BL/6 males and females were also assessed. We found that inactivation of MHCII, TSLPR and TLR4 had an overall similar impact across all four models used, suggesting similarities in the immune mechanisms underlying each of them. The impacts of sex and CD1d inactivation showed some variation among models, preferentially affecting the responses to HDM or OVA, respectively, regardless of the protocols of sensitisation and challenge used in each case.

The clearest-cut results were observed in MHCII-KO mice. These mice lack conventional CD4+ T cells, whereas CD1d-restricted CD4+ T cells are reported to be present in normal numbers [25]. ILC2 expression of MHCII is also expected to be defective in these mice [26]. We observed that eosinophil accumulation and PAS-positive staining in each of the models used were strictly dependent on the presence of conventional CD4+ T cells. This observation clearly points to an essential role of CD4+ T cells, regardless of allergen or immunization protocol, in each of our models, and is consistent with published studies reporting a key role of CD4+ T cells in several models of airway inflammation [27–29]. Our data also suggest that in these models cytokine-producing ILC2 or NKT cells are not sufficient for a response but may cooperate with CD4+ T cells for their function [30, 31]. In contrast to eosinophil infiltration and PAS staining, cellular infiltration in the peribronchial and perivascular areas of the lung were variably affected, suggesting that these responses also rely, at least in part, on innate components [32].

The inactivation of TSLPR also had a considerable impact on airway inflammation, and substantially reduced airway eosinophils in all models. Reduced eosinophil responses could be fully rescued by adoptive transfer of in vitro-primed Th2 cells into TSLPR-KO hosts, suggesting that, at the challenge phase, the role of TSLP was predominantly on the Th2 population [33]. In contrast, the effects of TSLPR inactivation on PAS-positive staining in bronchioles were less marked compared to the effects on eosinophils, or the effects observed in MHCII-KO mice, except for the local HDM model. This observation differs from previous studies [33–35] which used BALB/c TSLPR-KO mice (vs. our C57BL/6) and OVA preparations of unknown endotoxin content (vs. our low-endotoxin OVA) to report that goblet cell hyperplasia was also decreased, perhaps suggesting that genetic background and/or allergen composition can affect the impact of TSLPR deficiency on specific responses.

TSLP is known to have an essential function in allergic airway inflammation. TSLPR-KO mice developed reduced allergic airway inflammation in an OVA model [34], and lung-specific overexpression of TSLP can induce spontaneous allergic airway inflammation in mice [35]. TSLP is produced by airway epithelium exposed to LPS or protease-containing allergens [35, 36], and acts on multiple immune populations. In addition to activating DC [23, 37, 38], TSLP is necessary for the survival and effector function of memory Th2 cells [33, 39, 40]. Together with IL-33, TSLP can also induce cytokine production by ILC2 [13], with Th2 cells and ILC2 both contributing to cytokine production during allergic airway inflammation [17].

The differential impact of TSLPR deficiency we observed on eosinophils vs. PAS-positive staining in mice sensitised systemically with OVA or HDM was very unexpected, but consistent across the three models. This observation raises interesting questions about a differential TSLPR requirement for increased eosinophils vs. mucus production in lung and airway, which are dependent on IL-5 and IL-13, respectively. As both of these responses are strictly dependent on conventional CD4+ T cells, as indicated by experiments in MHCII-KO mice, this result may suggest a heterogeneity in TSLP requirement by either CD4+ Th2 subsets producing IL-5 vs. IL-13, or the CD4+ Th2 vs. ILC2 populations that can produce these cytokines. Results in this paper showing that repeated OVA challenges tend to decrease eosinophil numbers while increasing the percent of PAS-positive staining in bronchiole epithelium might also suggest a similar possibility. Studies in mice where the TSLPR is conditionally inactivated in selected immune populations in lung will be necessary in order to address these questions. Interestingly, decreased eosinophils and PAS-positive cells were both observed after i.n. HDM immunisation, which is consistent with a stronger dependence of this model on local innate immune mechanisms [9]. Overall, the results of these experiments are consistent with the recognized role of TSLP in supporting both innate and adaptive immune responses to allergens. Importantly, anti-TSLP has proven effective in ameliorating asthma symptoms in patients [41, 42], a result that is consistent with the essential role of TSLP in maintaining memory Th2 populations in vivo [33, 39].

NKT cells are glycolipid-reactive, innate-like T cells that can mediate allergic airway inflammation in the absence of conventional CD4+ T cells [43] and are necessary for airway inflammation and hyper-reactivity in an OVA model [44]. In contrast, the role of NKT cells in HDM models has not been examined. Unlike OVA, which does not contain NKT ligands, allergens such as HDM [16] and pollens [16, 45] are reported to contain glycolipids that induce NKT cell activation and production of IL-4 and IL-13. Consistent with previous reports, we observed that CD1d-KO mice, which lack NKT cells, generated impaired airway eosinophilia and PAS staining in OVA models, which were partially compensated by multiple airway challenges. In contrast, lack of NKT cells had no significant impact on the two models of HDM response, one of which used the same i.p. route of sensitisation and alum adjuvant also employed in the OVA models. This observation may suggest a role of NKT cells in amplifying responses to antigens that do not effectively engage innate immune mechanisms, such as the low-endotoxin OVA preparations used in our studies. However, the mechanism by which NKT cells might contribute to the OVA response measured here, the endogenous or exogenous source of potential NKT cell ligands in this model, as well as the role of NKT cells in clinical disease, remain poorly understood. Initial reports of a major contribution of NKT cells to allergic airway inflammation in patients [46] have been questioned [47]. The subsequent description of multiple NKT subsets with varying cytokine profiles [48] further adds to the complexity of this question.

In contrast to the clear impact of defective MHCII or TSLPR expression on airway eosinophilia, we found that responses in TLR4-KO mice were, for the most part, similar to the responses in wild-type C57BL/6 mice. The response to OVA was weakly to moderately stronger in TLR4-KO mice than in C57BL/6 mice, which might be due to the reported capacity of endotoxin to suppress priming to allergic airway inflammation [49]. In contrast to OVA, the response to HDM was not affected by TLR4 inactivation. Since the HDM protein Der p 2 is a known MD-2 mimic that facilitates signalling through TLR4 [12], and as such it is reported to exacerbate airway allergic responses via interaction with airway epithelial cells [36], this observation might suggest a low Der p 2 content in our HDM preparations [50]. Alternatively, other immunologically active components of HDM such as proteases, β-glucans and chitins [51] may be compensating for the loss of TLR4-dependent signalling in our model. While the impact of TLR4 inactivation on the models used here was unremarkable, it is important to note that TLR4 ligands have been shown to play important functions in other models of allergic response. Allergic conjunctivitis to Short Ragweed pollen [52] required TLR4 to drive the production of TSLP/OX40L and the priming of a productive Th2 response. Low endotoxin content in OVA and Cockroach extract preparations increased airway inflammatory responses [8, 53], and co-operated with Proteinase-activated receptor-2 (PAR_2_) signalling in inducing allergic sensitisation [53]. These studies highlight the multiple mechanisms through which allergens can engage with the immune system.

Finally, we report that C57BL/6 females develop stronger airway eosinophilia compared to male mice, but only in selected models. Intriguingly, the difference was strongest in the systemic HDM model, where eosinophil numbers are high, compared to the local model in which the weaker response might have been expected to be more dependent on cooperation with ILC2 [24]. Our results may also suggest that the impact of androgens and ILC2 in airway inflammation models may depend in part on the mouse colony and/or strain, as well as other properties of the allergen used.

A perhaps unexpected result from our study is the overall similarity of the results obtained using the i.n. HDM sensitisation model to results in other models using i.p. sensitisation with allergen in alum adjuvant. Whereas i.p. sensitisation with adjuvant is clearly not a physiological model of allergen exposure, the impact of KO mutations in MHCII, TSLPR, CD1d, and TLR4 on these artificial models was not dissimilar from the impact observed after i.n. sensitisation, suggesting that, regardless of the priming route, all these models essentially measure the activity of a population of effector Th2 cells with similar activation requirements. In this respect, it is also important to note that the natural route of airway allergen sensitisation in humans remains unknown, and may not necessarily be via the airway, with sensitisation via the skin remaining a realistic possibility [54].

While the innate properties of allergens clearly have an impact on their immunogenicity, and may have a stronger influence on inflammation than described here if used in settings of low-level or chronic allergen exposure, in our experiments they appeared mostly insufficient to directly drive inflammation independently of conventional CD4+ T cells. The study of such innate responses mostly requires tailored models in which specific allergens are used in high amounts and/or after careful purification to preserve their innate properties [50]. A relevant example is the powerful fungal airway allergen *Alternaria alternata* [15], which is itself a trigger of innate allergic responses [15] but, similar to IL-33, can also prime adaptive T cell responses in mice [55, 56] and humans [57]. It is also of interest that IL-33, which is essential for the innate function of *Alternaria* and many other environmental allergens [10], is also induced in macrophages by treatment with alum [58], and is rapidly produced in the peritoneum after i.p. injection of alum adjuvant [59]. Studies to examine lung ILC2 after i.p. alum injection may help establish whether the systemic and local priming of allergic immune responses in models of allergic airway inflammation such as those used here may involve common innate mechanisms.

## Conclusions

This work highlights the overall similarities and subtle differences in the immunological pathways that underlie murine allergic airway inflammation induced by different allergens and using different sensitisation protocols. This information may provide a useful basis for selecting experimental models for the study of allergic airway disease.

## Methods

### Mice

The following mouse strains were used: C57BL/6 J (originally from Jackson Laboratory, Bar Harbor, Maine, USA), TSLP-receptor (R) KO [60], MHCII-KO [22], CD1d-KO [61], TLR4-KO [62] and OTII [63]. Mice were bred by brother x sister mating and maintained in specific pathogen-free conditions at the Malaghan Institute of Medical Research, Wellington, NZ, with water and food ad libitum. Mice were age and sex-matched within experiments and used when 6–12 weeks old. Mice were euthanised for sample collection at the end of experiment by intraperitoneal injection of a high dose of Ketamine+Xylazine (300 and 9 mg/Kg, respectively). All experimental procedures were approved by the Victoria University of Wellington Animal Ethics Committee and carried out according to Institutional guidelines. No adverse effects were observed during this study.

### Allergic airway inflammation

Naïve mice were randomly assigned to control or experimental groups and sensitised i.p. with 200 μl Alu-S-Gel (1.3%) (Serva, Heidelberg, Germany) containing 2 μg OVA Grade V, (Sigma-Aldrich, Saint Louis, MO, USA), 40 μg *D. pteronyssinus* soluble extract (Greer Labs, Lenoir, NC, USA) or PBS (Gibco, Carlsbad, CA, USA). One hundred micrograms *D. pteronyssinus* crushed bodies (Greer Labs) were administered in 50 μl PBS i.n. to anaesthetised mice; HDM extract was also used in this model but gave very low eosinophil responses (not shown) and was not used further. HDM models were carried out using only female mice except for Fig. 6. For the adoptive transfer model, 5 million OTII Th2 cells were generated by co-culturing lymph node cells from OTII mice with LPS-activated C57BL/6 bone marrow-derived DC, generated as in [64], at an 8:1 ratio in the presence of 60 ng/ml IL-4, 20 ng/ml IL-2, and OVA_323–339_ peptide (ISQAVHAAHAEINEAGR) in 6 well plates. IL-4 and IL-2 were replenished on day 2 and 4. In vitro-activated CD4+ T cells were harvested on day 5 and their phenotype was checked by flow cytometry. Resulting cells were Vα2^+^Vβ5^+^CD62L^lo^CD69^hi^CD44^hi^ as described [5]. Five million cells were injected through the lateral tail vein into recipient mice (C57, CD1d-KO or TSLPR-KO), and mice were challenged i.n. 1 day after cell transfer.

For i.n. challenges, mice were anaesthetised with Ketamine+Xylazine at 100 and 3 mg/Kg, respectively, and 100 μg endograde OVA (Hyglos GmbH, Bernried, Germany), 100 μg HDM soluble extract or 25 μg HDM crushed bodies was administered dropwise into one nostril in 50 μl sterile PBS. The distribution of the i.n.**-**instilled solution was checked in preliminary experiments where mice were given coloured tracers. They were found to include lower airway and lung, although the lung was not uniformly involved. BAL was collected by flushing 1 ml of PBS through the lungs thrice. After red blood cell lysis, samples were processed for flow cytometry and counted using Accucount beads (Spherotech, Green Oaks, IL, USA).

### Flow cytometry

Antibody staining was performed in FACS Buffer (PBS with 2% FCS, 2 mM EDTA and 0.01% NaN3) using the following antibodies: anti-(a) CD11c-BV650, aMHCII (I-A/I-E)-Pacific Blue, aCD3-Pe-Cy7, aGr-1-AF647, aCD69-PE and aVα2-APC were from Biolegend (San Diego, CA, USA); aCD62L-PE-Cy7 and aCD44-APC-eFluor780 were from eBioscience (San Diego, CA, USA); aCD40-PE, aCD86-FITC, aVβ5.1/5.2-FITC, aNK1.1-PE, aSiglecF-PE-CF594, aCD19-APC-H7 and aCD4-BV605 were from Becton Dickinson (Franklin Lakes, NJ, USA); while aCD8-FITC and aCD16/32 hybridoma supernatant were prepared in house. Data were acquired on a LSRII SORP (Becton Dickinson, San Jose, CA, USA) or LSR Fortessa SORP (Becton Dickinson), and analysed using FlowJo Software v 9.9 (FlowJo LLC, Ashland, OR, USA). 4’,6-Diamidino-2-Phenylindole, Dihydrochloride (Molecular Probes, Eugene, OR, USA) was used to exclude dead cells.

### Histology

Lungs were harvested and fixed in 10% neutral buffered formalin (Sigma-Aldrich), cut in 5 μm sections on the coronal plane and stained with haematoxylin and eosin or Alcian Blue-Periodic Acid Schiff (PAS). Peribronchial and perivascular inflammation was scored by a blinded operator using the following criteria: (0), no peribronchial or perivascular infiltrates; (1), 1–2 centrally located microscopic foci of inflammatory infiltrates; (2), a dense inflammatory infiltrate in a perivascular or peribronchial distribution originating in the center of the lung and extending along the vessels or bronchi into the middle third of the lung parenchyma; (3), perivascular or peribronchial infiltrates extending to the periphery of lung and approaching the visceral pleura. To quantify AB-PAS staining, we used a quantitative and objective method in which 3 micrographs per lung were taken using an Olympus BX51 compound microscope at 20x magnification, and processed using an Image J macro to calculate the percent PAS-positive staining in the total bronchiole epithelial area excluding airspace (Additional file 1A).

### Statistics

All bar graph data are shown as mean +/− Standard Error of the Mean (SEM), excepting inflammation scores, which are expressed as median +/− interquartile ranges. All statistical analyses used a Mann-Whitney non-parametric *t-*test; *p* values lower than 5% were considered significant. Prism 7 for MAC OS X (San Diego, CA, USA) was used for all analyses.

## Additional files

Additional file 1: **Table S1.** Pdf file showing cell counts for neutrophils, T cells and alveolar macrophages relative to Fig. 1. (PDF 84 kb)

Additional file 2: **Figure S1.** Pdf file illustrating the quantification of AB-PAS-positive staining in airway epithelium using FIJI software, and the results of such quantification for the experiments in Fig. 1. (PDF 1970 kb)

Additional file 3: **Table S2.** Pdf file showing cell counts for neutrophils, T cells and alveolar macrophages relative to Figs. 2, 3, 4 and 5. (PDF 117 kb)

## Abbreviations

BAL: Broncho-alveolar lavage
DC: Dendritic cell/s
HDM: House dust mite
i.n.: intranasal/ly
IL: Interleukin
ILC2: Type-2 innate lymphoid cell/s
KO: Knockout
MHCII: Major Histocompatibility Complex Class II molecule
NKT: Natural Killer T (cell)
OVA: Chicken ovalbumin
PAS: Alcian Blue-Periodic Acid Schiff stain
PBS: Phosphate-buffered saline
SEM: Standard Error of the Mean
TLR: Toll-like receptor/s
TSLP: Thymic stromal lymphopoietin
TSLPR: TSLP receptor
WT: Wild-type
