# Supplementary material for: Similar immune mechanisms control experimental airway eosinophilia elicited by different allergens and treatment protocols
Source: BMC Immunol. 2019 Jun 4;20:18. doi: 10.1186/s12865-019-0295-y (PMC6549380; doi:10.1186/s12865-019-0295-y)
Supplement: Supplementary file 1 — Table S1. Pdf file showing cell counts for neutrophils, T cells and alveolar macrophages relative to Fig. 1. (PDF 84 kb) [file 12865_2019_295_MOESM1_ESM.pdf]

Additional file 1, *Hyde et al.*

Table 1: Cell counts for neutrophils, T cells and alveolar macrophages.

	**Neutrophils (x 10^3)**		**T cells (x 10^3)**		**Alveolar Macrophages (x 10^3)**	
	C57BL/6	PBS	C57BL/6	PBS	C57BL/6	PBS
**Acute OVA**	14.4 ± 1.7**	10.9 ± 3.2	36.8 ± 3.8****	6.2 ± 2.0	24.9 ± 1.8**	31.3 ± 2.1
**Repeat OVA**	5.7 ± 1.7****	0.2 ± 0.1	29.5 ± 2.9****	2.7 ± 0.6	27.6 ± 2.3	25.1 ± 1.2
**Systemic HDM**	5.3 ± 0.8****	1.0 ± 0.5	52.3 ± 8.6****	3.4 ± 1.1	19.7 ± 1.9***	33.6 ± 3.9
**Local HDM**	29.7 ± 5.1****	10.1 ± 1.5	21.9 ± 2.8****	3.1 ± 0.7	33.0 ± 4.4*	38.0 ± 3.4

C57BL/6 mice were immunized and challenged with OVA or HDM as in Figure 1; PBS mice were mock-immunized and challenged with OVA or HDM. Cell count data refer to the same experiments shown in Figure 1. Mean ± SEM is shown in all cases. P values refer to the comparison of allergen-sensitised and mock-sensitised mice that received allergen challenge. ***, p<0.001; **, p<0.01; *, p<0.05.
