# Supplementary material for: Similar immune mechanisms control experimental airway eosinophilia elicited by different allergens and treatment protocols
Source: BMC Immunol. 2019 Jun 4;20:18. doi: 10.1186/s12865-019-0295-y (PMC6549380; doi:10.1186/s12865-019-0295-y)
Supplement: Supplementary file 2 — Figure S1. Pdf file illustrating the quantification of AB-PAS-positive staining in airway epithelium using FIJI software, and the results of such quantification for the experiments in Fig. 1. (PDF 1970 kb) [file 12865_2019_295_MOESM2_ESM.pdf]

Additional file 2, *Hyde et al.*

Quantification of AB-PAS-positive staining in airway epithelium using FIJI software, and results of such quantification for Figure 1.

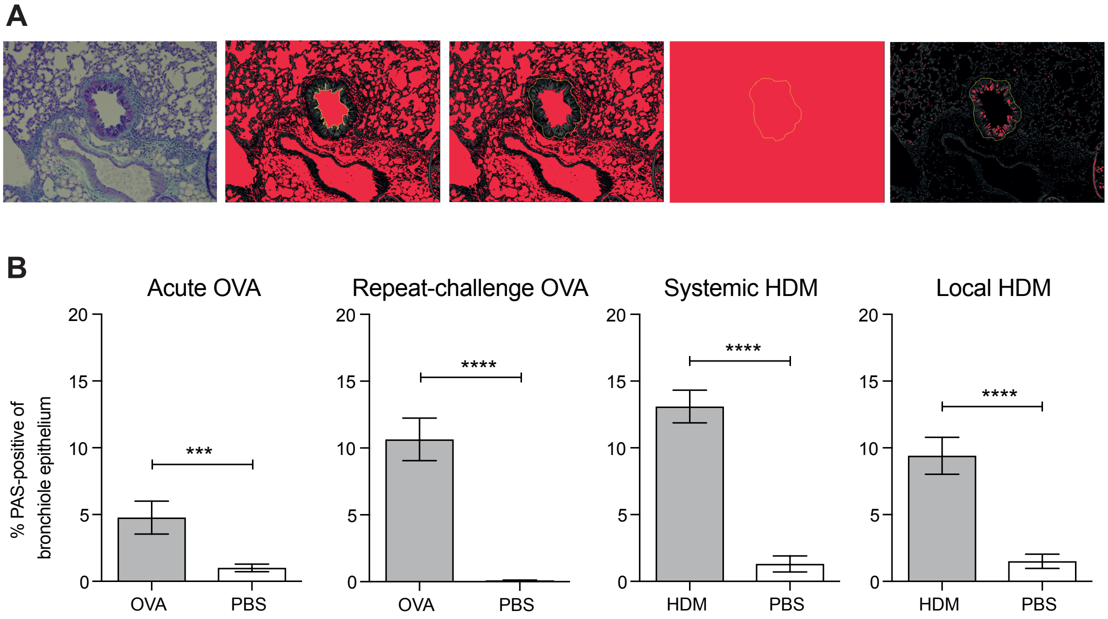

(A) Mucus staining was quantified as the proportion of AB-PAS-positive staining in the airway bronchiole epithelium area. Using FIJI software to enhance contrast of micrographs, the outer and inner perimeters of bronchioles were marked and the area calculated. The area of bronchiole epithelium was calculated by subtracting the area within the inner perimeter of the bronchiole from the total area within the outer perimeter of the bronchiole. Contrast was then adjusted so that only AB-PAS positive staining was highlighted in red and the area of red staining was divided by the area of bronchiole epithelium. (B) The area of AB-PAS-positive staining in bronchiole epithelium of C57BL/6 mice for each of four models of allergic airway inflammation in Figure 1 is shown. Bar graphs show mean ± SEM; ****, p<0.0001; ***, p<0.001.
