# Supplementary material for: Similar immune mechanisms control experimental airway eosinophilia elicited by different allergens and treatment protocols
Source: BMC Immunol. 2019 Jun 4;20:18. doi: 10.1186/s12865-019-0295-y (PMC6549380; doi:10.1186/s12865-019-0295-y)
Supplement: Supplementary file 3 — Table S2. Pdf file showing cell counts for neutrophils, T cells and alveolar macrophages relative to Figs. 2, 3, 4 and 5. (PDF 117 kb) [file 12865_2019_295_MOESM3_ESM.pdf]

Additional file 3, *Hyde et al.*

Table 2: Cell counts for neutrophils, T cells and alveolar macrophages.

		**Neutrophils (x 10^3)**			**T cells (x 10^3)**			**Alveolar Macrophages (x 10^3)**		
		C57BL/6	KO	PBS	C57BL/6	KO	PBS	C57BL/6	KO	PBS
**MHCII KO**	Acute OVA	8.9 ± 2.8	4.5 ± 2.6**	0.5 ± 0.3	43.3 ± 6.6	9.5 ± 2.7****	2.0 ± 0.3	18.1 ± 2.0	19.1 ± 2.0	23.8 ± 1.7
	Repeat OVA	2.0 ± 0.6	0.5 ± 0.1***	0.1 ± 0.1	25.0 ± 1.9	10.1 ± 3.9**	2.9 ± 0.9	27.8 ± 4.4	14.5 ± 1.2	23.3 ± 1.5
	Systemic HDM	8.4 ± 1.6	0.9 ± 0.4****	0.2 ± 0.04	80.9 ± 24.6	3.1 ± 1.0*	2.3 ± 0.7	11.6 ± 2.2	24.0 ± 3.2**	24.6 ± 4.8
	Local HDM	26.5 ± 3.7	34.4 ± 4.9	14.7 ± 3.1	18.5 ± 2.5	13.9 ± 4.3	4.2 ± 1.7	54.5 ± 11.8	37.7 ± 3.2	55.0 ± 7.4
**TSLPR KO**	Acute OVA	6.4 ± 2.1	0.4 ± 0.1****	0.4 ± 0.2	15.8 ± 1.8	4.5 ± 1.2****	1.3 ± 0.3	24.9 ± 2.3	46.0 ± 6.1***	34.0 ± 5.0
	Repeat OVA	7.2 ± 3.6	0.7 ± 0.3***	0.2 ± 0.1	23.0 ± 4.1	10.0 ± 2.4**	3.8 ± 1.6	29.9 ± 4.8	26.3 ± 2.3	24.5 ± 2.5
	Systemic HDM	4.7 ± 1.1	0.3 ± 0.1***	0.1 ± 0.0	60.0 ± 11.6	11.7 ± 2.6****	2.2 ± 0.7	23.7 ± 3.8	24.3 ± 1.9	40.4 ± 9.7
	Local HDM	20.1 ± 5.1	10.1 ± 1.6	4.9 ± 0.8	14.3 ± 2.6	12.3 ± 2.74	1.5 ± 0.6	24.1 ± 3.2	25.2 ± 2.6	29.9 ± 1.9
**CD1d KO**	Acute OVA	9.1 ± 2.0	3.4 ± 1.2*	0.6 ± 0.3	48.0 ± 6.4	16.3 ± 2.3***	2.3 ± 0.3	21.5 ± 2.6	23.1 ± 2.8	32.0 ± 3.4
	Repeat OVA	7.7 ± 3.3	1.9 ± 0.9*	0.2 ± 0.1	40.3 ± 6.5	29.5 ± 3.8	1.3 ± 0.2	25.2 ± 2.3	25.4 ± 2.5	27.6 ± 2.1
	Systemic HDM	6.9 ± 1.2	2.8 ± 0.8**	2.0 ± 1.0	23.8 ± 6.5	30.2 ± 5.9	5.5± 2.3	19.5 ± 3.3	29.8 ± 5.7	31.6 ± 4.8
	Local HDM	42.3 ± 10.8	32.4 ± 6.0	9.6 ± 1.9	30.1 ± 5.5	27.0 ± 4.9	2.7 ± 0.7	43.1 ± 9.6	39.8 ± 5.2	40.8 ± 6.4
**TLR4 KO**	Acute OVA	3.6 ± 1.0	13.4 ± 4.7**	0.3 ± 0.1	23.2 ± 4.5	38.4 ± 6.1**	1.4 ± 0.3	30.4 ± 3.8	32.8 ± 3.7	36.1 ± 5.0
	Repeat OVA	2.5 ± 0.7	2.8 ± 0.5	0.1 ± 0.1	17.2 ± 1.9	38.1 ± 5.9***	2.7± 0.7	33.2 ± 5.1	40.0 ± 8.1	26.3 ± 2.4
	Systemic HDM	1.5 ± 0.6	5.7 ± 1.9*	0.1 ± 0.0	18.8 ± 3.6	17.7 ± 3.3	1.4 ± 0.3	20.4 ± 2.6	15.0 ± 2.0	26.3 ± 1.9
	Local HDM	26.5 ± 3.7	18.9 ± 3.0	14.7 ± 3.1	18.5 ± 2.5	15.5 ± 2.6	4.2 ± 1.7	54.5 ± 11.8	38.1 ± 5.9	55.0 ± 7.4

C57BL/6 and the indicated strains of KO mice were immunized and challenged with OVA or HDM as in Figure 1; PBS mice were mock-immunized and challenged with OVA or HDM. Cell count data refer to the same experiments shown in Figures 2-5. Mean ± SEM is shown in all cases. P values refer to the comparison of WT and KO mice with allergen sensitisation and challenge. ***, $p<0.001$; **, $p<0.01$; *, $p<0.05$.
